# Delayed presentation of spinal cord injury without radiographic abnormality (SCIWORA): a case report

**DOI:** 10.11604/pamj.2023.45.160.23467

**Published:** 2023-08-15

**Authors:** Hedhli Hana, Hedia Gnena, Othmani Safia, Ben Kaddour Rym, Asma Jendoubi, Zoubli Aymen, Fares Ouni, Sarra Jouini

**Affiliations:** 1 Emergency Department, Charles Nicolle’s Teaching Hospital, Tunis, Tunisia,; 2 Faculty of Medicine of Tunis, University of Tunis, El Manar, Tunisia

**Keywords:** trauma, spinal cord injury, magnetic resonance imaging, degenerative disc disease, case report

## Abstract

Spinal Cord Injury without Radiographic Abnormality (SCIWORA) is an unprecedented event to occur in adults but may lead to serious complications including permanent neurological impairments and death. In this article, we report a case of a 60-year-old male presenting to the emergency for a head and neck trauma after a motorbike accident, who reported only a neck ache and a normal primary neurological exam. The evolution was marked by the occurrence of paraplegia with a diminished sphincter tone and hypoesthesia beneath the T12 dermatome level after six hours, confirmed by the Magnetic Resonance Imaging (MRI). He underwent a deferred laminectomy within 30 days after receiving methylprednisolone, with partial neurological improvement after two months. An early recognition of the diagnosis was challenge for the emergency physician, given the wide variability of clinical presentations. Magnetic resonance imaging (MRI) is a key examination to guide the diagnosis and the management of these patients.

## Introduction

Post-traumatic spinal cord injury may lead to a pernicious condition for the patients and their social circle. In most cases, it is due to fractures and dislocations that are depicted by means of radiography or Computed Tomography (CT) [1]. The SCIWORA (Spinal cord injury without radiographic abnormality) defines spinal cord lesions that are discovered in Magnetic Resonance Imaging (MRI), but that does not show any post-traumatic bone lesions in plain radiographs and tomography [2]. The debate is still active around the term “Adult SCIWORA” mainly about the uncertainty of classification and common comorbidity, that is why SCIWORA still represents a diagnostic challenge for emergency physicians. We report a case of an adult of a 60-year-old patient to illustrate the diagnostic approach of SCIWORA and optimize timely diagnosis and management of this trauma.

## Patient and observation

**Patient information:** a 60-year-old male presented to the emergency department for head and neck trauma after a motorbike accident, high-speed skidding and falling with head-level reception, without using a head helmet for protection.

**Clinical findings:** on admission, he was conscious and had normal vital signs. He complained of neck pain, the primary physical neurological examination has not revealed any abnormalities. Considering high-velocity injury according to the vittel criterias, the patient underwent a body scan, showing only degenerative disc disease in the spine. We have not detected any fractures or dislocations. Within 6 hours of monitoring, the patient has developed paraplegia with a diminished sphincter tone and hypoesthesia below the T12 dermatome level. Following the 2019 revised ASIA impairment scale, the neural injuries were classified as a sensory level at T12 and complete, thus compatible with a grade A spinal cord injury.

**Figure 1:**
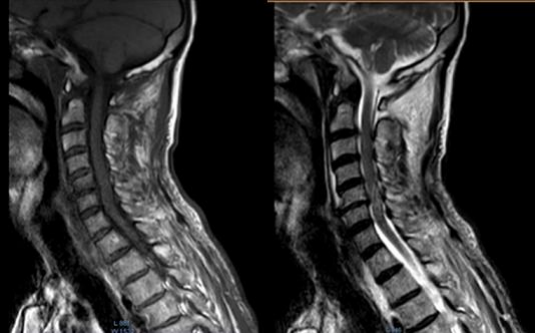
T1 and T2 weighted magnetic resonance imaging: Images show C4-C5 and C5-C6 degenerative disc disease and spinal cord swelling

**Figure 2:**
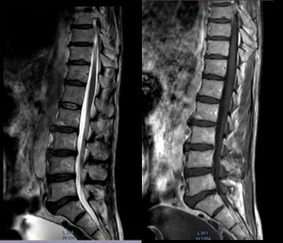
T1 and T2 weighted magnetic resonance imaging; images show expanded lumbar disc degenerative disease

**Figure 3:**
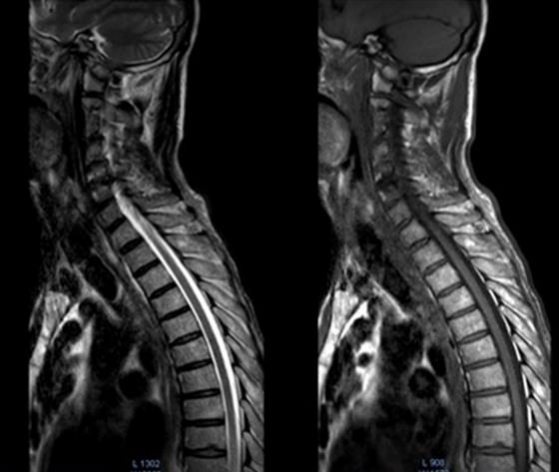
T1 and T2 weighted magnetic resonance imaging; images show a normal thoracic marrow

**Diagnostic assessment:** given the discrepancy between clinical presentation and the absence of underlying cause demonstrated by conventional CT, an MRI was performed. The MRI revealed cervical degenerative disc disease mainly at C5-C6 and C4-05 levels with disco osteophytes filling the anterior epidural space and coming into contact with the marrow with a posterior T2 hyper signal lesion (Figure 1). The lumbar spine also suffered from expanded degenerative disc disease without marrow abnormality (Figure 2). The thoracic marrow was also normal (Figure 3).

**Diagnosis:** the diagnosis of SCIWORA was finally confirmed based on the clinical presentation and magnetic resonance imaging findings.

**Therapeutic interventions:** the patient was initially hospitalized in the care unit of the emergency department to continue the monitoring for parameters and he received methylprednisolone. He did not benefit from either spinal traction or surgical decompression.

**Follow-up and outcome of interventions:** two days later, the evolution was marked by the occurrence of paresis of the two upper limbs with a sensory level at C5 without respiratory involvement, hence he was transferred to the orthopedic department. He underwent a deferred intervention (laminectomy of medical supervision) regarding the no improvement of the neurological status within 30 days. No complications were observed in the postoperative period.

**Follow-up and outcomes:** the patient was transferred to a Rehabilitation Medicine Department for subsequent treatment. The follow-up of the patient after two months of the treatment showed a partial neurological improvement.

**Patient perspective:** he no longer has hypoesthesia and began to move his lower limbs.

**Informed consent:** we obtained informed consent from the patient to use the images in this case report.

## Discussion

In 1982, Pang and Wilbarger were first to define the acronym SCIWORA in a series of 24 children who have been diagnosed with cervical traumatic myelopathy without any radiological evidence of fractures neither dislocations [3]. It is more typically observed in the pediatric group and entails particularly the cervical spine instead of the thoracic and lumbar spine [4]. A recent systematic review, Carroll T *et al*. [5] established that among 386 pediatric cases, cervical spine was concerned in 87% of the cases, thoracic spine was concerned in 9.5%, lumbar spine was concerned. In 1.5%, and in 2% the spinal cord damage bridged the cervical and thoracic levels. Interestingly, the lower cervical spine was more prone to SCIWORA in older children Then in younger children, according to several studies. This finding is supported by the fact that the fulcrum of motion is on the lower tiers of the cervical spine (between C5 and C6) in adolescents and adults and this is well suited with our patient who had a swelling spine particularly at the level of C5-C6 [6]. SCIWORA in adults is a sparse phenomenon. In a series of 472 adult patients presenting with a spinal cord injury, Tewari MK *et al*. [7], only 40 were diagnosed with SCIWORA. It occurs commonly in males, with a peak in their productive ages. Mobility and vulnerability of the cervical spine to trauma promotes damage.

According to a recent review, Atesok K *et al*. [8] confirmed that the evidence from the adult population indicates that thoracolumbar spine can also be inculpated with SCIWORA but only limited to sporadic case reports. SCIWORA might be explained by various mechanisms including extrinsic cord injury from hyperextension, spinal cord traction damage due to hyperflexion, and parenchymal cord damage due to edema or vascular injury [9]. The two-hit hypothesis might offer an explanation for the delayed cord damage in patients with SCIWORA, as seen in our case report with occurrence of neurological abnormalities after six hours. Furthermore, neurological deficits might worsen or appear several days or even weeks later, due to edema, or a developing hematoma around the cord or second-hit phenomenon. In point of fact, beyond the primary injury caused from direct impact, a subsequent secondary injury to the spinal cord parenchyma from complex cellular-level reactions to the primary injury could aggravate the clinical evolution of the patients. Our patient benefited initially from non-surgical treatment for 30 days and had Methylprednisolone as per North American Society for Cardiac Imaging III criteria. He was then operated by orthopedic surgeons, given that he didn´t show any neurological improvement. In fact, treatment methods differ from one institution to another; and until this day no definitive protocol has been established. Non-surgical treatment is reasonable considering that post-traumatic spinal cord damage results from both primary (direct impact) and secondary mechanisms (mainly an inflammatory response that is prolonged for days till weeks after the injury). Hence, intravenous methylprednisolone can be beneficial in improving outcomes and preventing secondary damage but may require further investigation and evidence.

According to Atesok *et al*. [8] for patients with normal or pure intraneural MRI lesions (contusion without compression). Surgical treatment is not recommended in SCIWORA despite the neurological status. If the MRI depicts ligamentous injury, instability, spinal cord compression associated with worsened, or unimproved neurological findings, surgical decompression should be indicated. Till 2018, we do not dispose of controlled trials comparing in SCIWORA patient’s outcomes of nonsurgical versus surgical treatment.

## Conclusion

SCIWORA is a challenge for clinicians to identify spinal cord injuries if high clinical suspicion in patients with normal radiographs and CT. For this reason, the MRI appears as the optimal mode of imaging in trauma patients that can help physicians in understanding of SCIWORA syndrome. Medical treatment can be sufficient, and there is no consensus for the operative treatment which is necessary for selected patients. Regarding the male and young population concerned by SCIWORA, outcomes can be devastating either for the patient himself or for the society.
